# [^18^F]florbetapir PET for early detection of amyloidosis in patients with hereditary transthyretin amyloidosis polyneuropathy

**DOI:** 10.1016/j.gendis.2025.101710

**Published:** 2025-06-07

**Authors:** Yanyan Kong, Lei Cao, Boyan He, Zhongwen Zhou, Minmin Zhang, Qian Zhang, Qian Wang, Wei Wang, Haoxiang Zhu, Jianfei Xiao, Axel Rominger, Yihui Guan, Haibo Tan, Ruiqing Ni

**Affiliations:** a PET Center, Huashan Hospital, Fudan University, Shanghai 200235, China; b Institute for Regenerative Medicine, University of Zurich, Zurich 8952, Switzerland; c Department of Pathology, Huashan Hospital, Fudan University, Shanghai 200235, China; d Department of Nephrology, Huashan Hospital, Fudan University, Shanghai 200235, China; e Department of Hematology, Huashan Hospital, Fudan University, Shanghai 200235, China; f Department of Infectious Diseases, Huashan Hospital, Fudan University, Shanghai 200235, China; g Department of Nuclear Medicine, Bern University Hospital, Bern 3010, Switzerland; h Institute for Biomedical Engineering, University of Zurich & ETH Zurich, Zurich 8093, Switzerland

Systemic amyloidosis is a heterogeneous group of diseases characterized by localized or systemic deposition of insoluble extracellular fibrillary proteins in organs. Systemic amyloidosis is categorized by precursor protein, and the most common are immunoglobulin light-chain amyloidosis (AL) and transthyretin (TTR) protein produced predominantly in the liver (ATTR amyloidosis). ATTR amyloidosis frequently results from age-related misfolding of wild-type TTR and, less commonly, from misfolding of a variant TTR from an autosomal dominant mutation of the TTR gene.^1^ Hereditary ATTR (hATTR) is a progressive and potentially fatal disease with a heterogeneous clinical presentation; patients often develop a mixed phenotype of polyneuropathy (PN) characterized by sensory, motor, and autonomic neuropathy and/or cardiac amyloidosis.^1^ The survival rate varies widely, ranging from 3 to 15 years after diagnosis, depending on factors such as genetic mutation and clinical phenotype. It is extremely critical for the detection of amyloidosis at the earliest stage, when therapy is still effective before severe organ damage occurs.

Noninvasive imaging approaches, such as scintigraphy using the calcification detection tracer [^99m^Tc]3,3-diphosphono-1,2-propanodicarboxylic acid (DPD), [^99m^Tc]pyrophosphate, [^99m^Tc]hydroxymethylene diphosphonate (HMDP), and [^123^I]serum amyloid P component and cardiac magnetic resonance imaging, have been used to assist in the diagnosis of systemic amyloidosis.^2^ Positron emission tomography (PET) using [^18^F]NaF, a fibroblast activation protein inhibitor tracer, [^68^Ga]pentixa, [^64^Cu]fibrin-binding-protein, [^18^F]fluorodeoxyglucose, and amyloid imaging tracers [^18^F]flutemetamol, [^18^F]florbetapir (FBP), [^18^F]florbetaben, and [^11^C]PIB has been used in the clinical evaluation of systemic amyloidosis. These amyloid imaging tracers have demonstrated prognostic utility in detecting cardiac amyloidosis in patients with hATTR and AL, as well as in identifying pulmonary involvement in individuals with AL. [^18^F]FBP PET has been reported for the diagnosis and follow-up of AL patients with cardiac or multiorgan involvement and ATTR patients. [^11^C]PIB PET/computed tomography (CT) has been used for treatment monitoring with tafamidis in patients with ATTR. Moreover, [^18^F]florbetaben PET/CT is in a clinical trial for differential diagnosis among light chain cardiac amyloidosis, ATTR, and mimicking conditions.

In the present study, we assessed the utility of [^18^F]FBP imaging in detecting amyloidosis in hATTR-PN patients carrying p.H76R, p.A117S, p.K55N, or p.T69A mutations, for which no amyloid PET has been reported, as well as in the more common p.V50M mutation carriers. Seventeen participants, including 9 normal controls (aged 58.9 ± 7.9 years) and 8 patients with hATTR-PN (aged 55.4 ± 13.7 years), were recruited from Huashan Hospital in Shanghai, China, from 2022 to 2024 (demographics in Table S1). Genetic testing, biopsy, and biochemical assays of the patient's serum and urine were conducted. Whole-body PET/CT using [^18^F]FBP was performed, and amyloid deposition was assessed using maximum standard uptake value (SUV_max_) and target-to-background ratio (TBR) analyses with mediastinal blood pool as reference region (Fig. S2). The detailed methods are described in the supplemental file.

Among the 8 patients with hATTR-PN, 1 patient carrying p.H76R (c.227A > G), 2 patients carrying p.A117S (c.394G > T), 2 patients carrying p.V50M (c.148G > A), 2 patients carrying p.T69A (c.205A > G), and 1 patient carrying p.K55N (c.165G > C) were included. The clinical symptoms reported in the literature are consistent with these cases. One hATTR-PN (p.H76R) patient clinically manifested with first-degree atrioventricular block, cardiac radiofrequency ablation, prostatic hyperplasia, and pleural effusion. The two hATTR-PN (p.A117S) patients clinically manifested multiple sensorimotor peripheral neuropathies, with amyloidosis in the peripheral nerve plexus and heart and distal cold limbs. Two hATTR-PN (p.V50M) patients exhibited amyloidosis in the peripheral nerve plexus and sensorimotor peripheral impairment. One patient with p.T69A had amyloidosis in the peripheral nerve plexus with normal cardiac function (aged 45 years, female), whereas the other two had cardiac amyloidosis in the gastrointestinal system and sensorimotor peripheral impairment (aged 58 years, male). The hATTR-PN (p.K55N) patient clinically manifested with vitreous amyloidosis in both eyes. The levels of biochemical markers in the hATTR-PN groups are shown in Table S2.

All hATTR-PN patients (p.A117S, p.V50M, p.T69A, or p.K55N p.H76R mutation carriers) presented positive [^18^F]FBP PET results and diverse distribution patterns: p.A117S mutation carrier-positive uptake in the heart, kidneys, bone marrow (BM), fat, and muscles (Fig. 1A–C); p.T69A mutation carrier-positive uptake in the BM, stomach, pelvic floor fat, kidneys, and muscles (Fig. 1D–G); p.K55N mutation carrier-positive uptake in the vitreous body, heart, muscles, lungs, and BM (Fig. 1H–K); p.V50M mutation carrier-positive uptake in the kidneys and muscles (Fig. 1L, M); and p.H76R mutation carrier-positive uptake in heart, tongue, muscles, kidneys, BM, and fat (Fig. 1O–U). We further validated the accuracy of [^18^F]FBP PET findings via histological staining of amyloid deposits in biopsy tissues. Organ involvement by [^18^F]FBP was most observed in the muscle, BM, and kidney (>85%) of patients with hATTR-PN (Fig. S1). We detected a greater number of involved organs by SUV_max_ and visual analysis of [^18^F]FBP in hATTR-PN patients (p.A117S, p.V50M, p.K55N, p.T69A, and p.H76R) than in normal controls (*p* < 0.0001 and *p* < 0.0001, respectively; Fig. 1V). In addition, TBR analysis revealed greater [^18^F]FBP uptake in the liver and BM in hATTR-PN patients than in the NC group (*p* < 0.0001 and *p* = 0.0211, respectively; Fig. 1X). In hATTR-PN patients, a greater number and percentage of involved organs were detected by SUV_max_ and visual analysis compared with clinical evaluation, with no difference between SUV_max_ and visual analysis.

**Figure 1:**
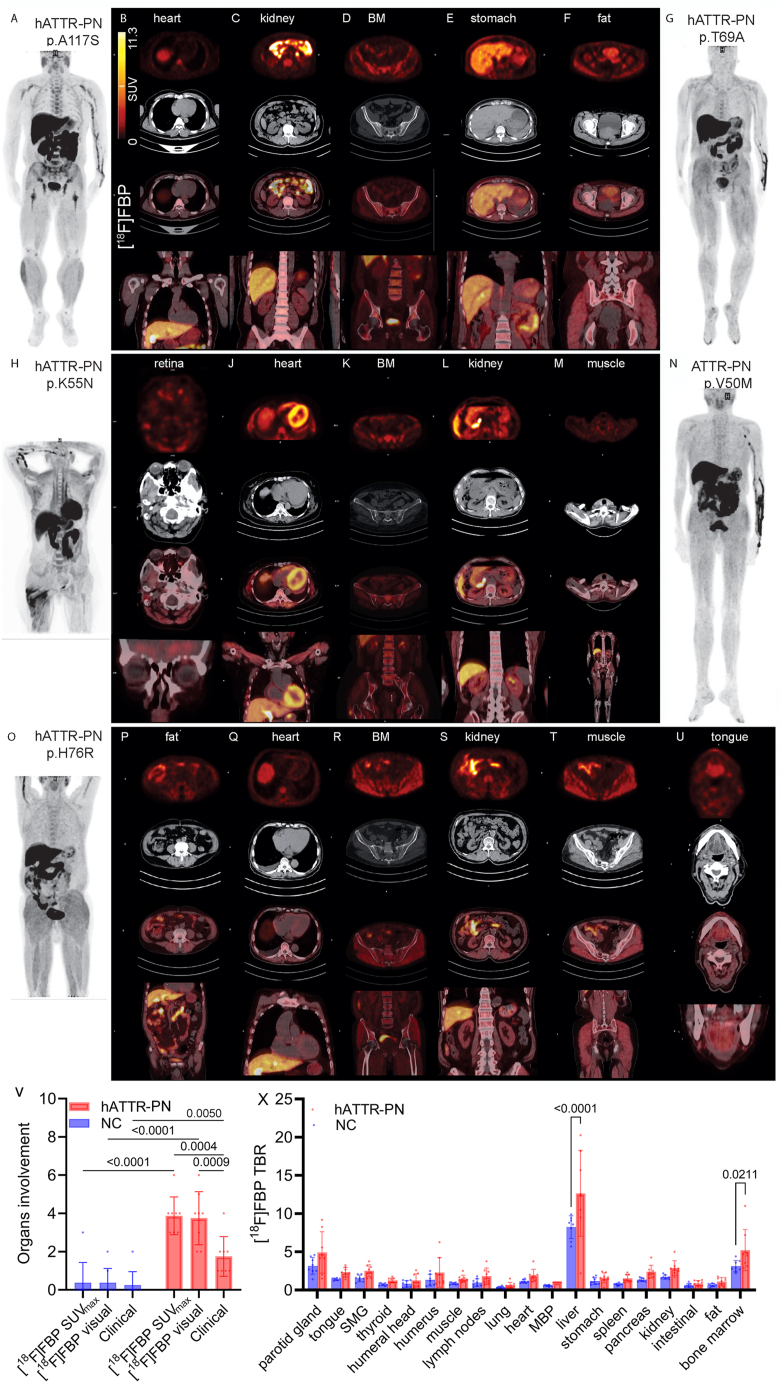
[^18^F]FBP imaging in hATTR-PN patients and normal controls for assessing organ involvement. **(A, G, H, N, O)** Maximum intensity projection (MIP) of [^18^F]FBP whole-body scans of hATTR-PN patients. Transaxial PET, CT, transaxial and coronal images of overlay images of the heart **(B)**, kidney **(C)**, and bone marrow **(D)** from a patient with hATTR-PN (p.A117S, aged 48 years old, male, A); stomach **(E)**, and fat in the pelvic floor **(F)** from a patient with hATTR-PN (p.T69A, aged 45 years old, female, G); retina **(I)**, heart **(J)**, and BM **(K)** from a patient with hATTR-PN (p.K55aN, aged 60 years old, female, H); and kidney **(L)** and muscle **(M)** from a hATTR-PN patient (p.V50M, aged 67 years old, male, N). and fat **(P)**, heart **(Q)**, kidney **(R)**, muscle **(S)**, and tongue **(U)** of a hATTR-PN patient (p.H76R, aged 82 years old, male, O). **(V)** Comparison between clinical assessment and visual and SUV_max_ analysis of [^18^F]FBP for organ involvement in NC (normal control) and hATTR-PN patients. **(X)** Regional uptake of [^18^F]FBP in NC and hATTR-PN patients by target-to-background ratio (TBR) analysis. hATTR, hereditary transthyretin amyloidosis; PN, polyneuropathy; MBP, Mediastinal blood pool; SMG, submandibular gland.

The most common genetic variants in hATTR worldwide are V30M (p.V50M) and T60A worldwide.^3^ Several PET studies have reported greater uptake in patients with hATTR-CA carrying V30M (p. V50M) than in healthy controls when [^18^F]FBP, [^18^F]flutemetamol,^4^ and [^11^C]PIB are used. Cerebellum and cerebral amyloid deposition in long-term hATTR V30M (p.V50M) patients has been previously reported using [^18^F]flutemetamol PET.^4^ In our study, no central nervous system involvement was observed in hATTR-PN (p.V50M) or hATTR-PN harboring other mutations. Positive [^11^C]PIB uptake has been reported in various organs of hATTR patients carrying G47R, T60A, or D18G mutations. The hATTR-PN p.A117S (A97S) mutation has been reported in a few studies in East Asian populations but has not been reported in Caucasians. hATTR-PN p.T69A (T49A) patients were reported in two studies from France and Italy, and hATTR-PN p.H76R (H56R) patients were reported once with geographic kindreds in the USA and were both reported for the first time in the East Asian population. Thus far, only one case report on an hATTR-PN p.K55N (K35N) patient with vitreous amyloidosis, whose clinical manifestations were similar to those in the present study, has been published.

Scintigraphy with [^99m^Tc]DPD and [^99m^Tc]HMDP was found to be suboptimal in TTR-cardiac amyloidosis patients with V30M (p.V50M) or p.F64L mutations but positive in, *e.g.*, E89Q, A36P, T49A, F33V, and I68L mutation carriers.^5^ These findings highlight the unique value of PET using amyloid tracers such as [^18^F]FBP in detecting amyloid deposits in patients with hATTR. Differences in the binding sites of amyloid PET tracers on amyloid-beta fibrils have previously been described in sporadic and autosomal dominant Alzheimer's disease. An *ex vivo* study showed that the amyloid tracer [^3^H]PIB and the flutemetamol analog cyano-flutemetamol were able to detect AL and ATTR amyloid deposits. Further study on the binding affinity of the amyloid tracer for ATTR fibrils with different mutations will be informative.

In conclusion, our findings suggest that [^18^F]FBP PET is a powerful noninvasive imaging tool for the early detection and characterization of amyloid deposition patterns in hATTR-PN patients carrying p.A117S, p.K55N, p.H76R, or p.T69A mutations. The ability of [^18^F]FBP PET to characterize amyloid deposition patterns in hATTR-PN patients with different mutations may guide personalized treatment decisions and monitor treatment response, such as the use of transthyretin gene silencing, liver transplantation, or pharmacotherapies. Early detection of amyloidosis in hATTR-PN patients via [^18^F]FBP PET is clinically critical, as it facilitates timely intervention before irreversible organ damage develops and enables the identification of amyloid deposition in organs typically inaccessible to biopsy.

## CRediT authorship contribution statement

**Yanyan Kong:** Writing – review & editing, Writing – original draft, Funding acquisition, Formal analysis, Data curation, Conceptualization. **Lei Cao:** Writing – review & editing, Methodology, Formal analysis. **Boyan He:** Writing – review & editing, Formal analysis, Data curation. **Zhongwen Zhou:** Methodology, Data curation. **Minmin Zhang:** Writing – review & editing, Methodology, Data curation. **Qian Zhang:** Writing – review & editing, Formal analysis, Data curation. **Qian Wang:** Writing – review & editing, Methodology, Formal analysis, Data curation. **Wei Wang:** Writing – review & editing, Formal analysis, Data curation. **Haoxiang Zhu:** Writing – review & editing, Formal analysis, Data curation. **Jianfei Xiao:** Writing – review & editing, Methodology, Formal analysis. **Axel Rominger:** Writing – review & editing, Writing – original draft. **Yihui Guan:** Writing – review & editing, Writing – original draft, Resources, Funding acquisition. **Haibo Tan:** Writing – review & editing, Writing – original draft, Data curation, Conceptualization. **Ruiqing Ni:** Writing – review & editing, Writing – original draft, Funding acquisition, Conceptualization.

## Funding

YK received funding from the National Natural Science Foundation of China (No. 82272108), the Natural Science Foundation of Shanghai (No. 22ZR1409200), China Scholarship Council (No. 202306100074) and the Shanghai Science and Technology Innovation Action Plan Medical Innovation Research Project (23Y11903200). YG received funding from the National Natural Science Foundation of China (No. 82071962). RN acknowledges the Swiss Center for Applied Human Toxicology (SCAHT AP22-01).

## Ethics declaration

This study was approved by the Institutional Ethics Review Board of Huashan Hospital, Fudan University (2022-535). Informed consent was obtained from all participants.
