# Supplementary material for: [18F]florbetapir PET for early detection of amyloidosis in patients with hereditary transthyretin amyloidosis polyneuropathy
Source: Genes Dis. 2025 Jun 7;13(2):101710. doi: 10.1016/j.gendis.2025.101710 (PMC12682007; doi:10.1016/j.gendis.2025.101710)
Supplement: Multimedia component 1 [file mmc1.docx]

**Supplementary files**

STable 1 **Demographics** of the involved organs in **hATTR-PN patients**

| **No** | **Sex** | **Age** | **Diagnosis** | **Involved organs (clinical)** |
| --- | --- | --- | --- | --- |
| 1 | M | 48 | hATTR-PN p.A117S c.349G>T | Nerve plexus, heart |
| 2 | M | 58 | hATTR-PN p.A117S c.349G>T | Nerve plexus, heart |
| 3 | F | 60 | hATTR-PN p.K55N c.165G>C | Vitreous body of both eyes |
| 4 | M | 75 | hATTR-PN p.V50M c.148G>A | Nerve plexus |
| 5 | M | 67 | hATTR-PN p.V50M c.148G>A | Nerve plexus |
| 6 | F | 45 | hATTR-PN p.T69A c.205A>G | Heart |
| 7 | M | 35 | hATTR-PN p.T69A c.205A>G | Nerve plexus |
| 8 | M | 82 | hATTR-PN p.H76R c.227A>G | Heart, gastrointestinal, fat |
| 9 | M | 52 | NC | / |
| 10 | M | 55 | NC | / |
| 11 | F | 68 | NC | / |
| 12 | F | 56 | NC | / |
| 13 | M | 57 | NC | / |
| 14 | M | 45 | NC | / |
| 15 | M | 64 | NC | / |
| 16 | M | 65 | NC | / |
| 17 | F | 68 | NC | / |

hATTR-PN, hereditary transthyretin amyloidosis-polyneuropathy;

.

STable 2 Analysis of biochemical markers

| Biochemical markers | hATTR-PN (n = 8) |
| --- | --- |
| Thyroid-stimulating hormone (mIU/L) (0.27-4.2) | 1.2±0.2 |
| Triiodothyronine (nmol/L) (1.3-3.1) | 1.4±0.2 |
| Thyroxine (nmol/L) 66-181 | 93.4±14.9 |
| Free triiodothyronine (pmol/L) 3.1-6.8 | 4.3±0.4 |
| Free thyroxine (pmol/L) 12-22 | 15.9±2.0 |
| Thyroglobulin antibody (U/ml) <115 | 47.2±43.8 |
| Thyroid peroxidase antibody (U/ml) <34 | 43.9±33.2 |
| Thyroglobulin (ng/ml) 3.5-7.7 | 9.3±7.3 |
| Thyrotropin receptor antibody (IU/L) <1.75 | 1.5±1.1 |
| AST (U/L) 9-50 | 20.1±4.5 |
| ALT (U/L) 15-40 | 22.8±2.7 |
| Glycated hemoglobin (HbA1c%) 4-6 | 5.4±0.3 |
| Blood sugar (mmol/L) 3.9-5.8 | 5.1±0.9 |
| γ-GT (U/L) 10-60 | 18.0±5.2 |
| ALP (U/L) 45-125 | 65.0±14.8 |
| Creatinine (μmol/L) 57-111 | 63.3±17.6 |
| Urinary protein (g/24 h) <0.15 | 0.1±0.0 |
| Total bilirubin (ng/ml) <26 | 7.4±1.8 |
| eGFR(ml/min) MDRD ≥90, | 132.1±21.7 |
| eGFR(ml/min) EPI ≥90 | 107.7±6.3 |
| NT-proBNP (pg/mL) <219.3 | 603.6±1045.5 |
| Myoglobin (ng/ml) 25-58 | 78.4±64.9 |
| CK-MB mass (ng/ml) ≤3.61 | 6.6±5.5 |
| Systolic blood pressure | 110.2±14.3 |
| diastolic blood pressure | 70.2±8.1 |
| IgM (g/L) 0.3-2.2 | 1.0±0.1 |
| IgG (g/L) 8.6-17.4 | 15.4±0.6 |
| IgA (g/L) 1-4.2 | 3.7±0.4 |
| sFLC k  (mg/L) 6.7-22.4 | 17.8±2.8 |
| sFLC λ  (mg/L) 8.3-27 | 20.9±4.9 |
| sFLC k/λ  0.31-1.56 | 0.9±0.2 |
| κ (g/L) 1.7-3.7 | 3.1±0.5 |
| λ (g/L) 0.9-2.1 | 1.7±0.2 |
| κ/λ 1.35-2.65 | 1.8±0.1 |
| β2 microglobulin (mg/l) 0.7-1.8 | 1.9±0.4 |
| Urinary -κ- light chain (mg/L) <7.5 | 10.7±5.1 |
| Urinary-λ-light chain (mg/L) <4.1 | 3.9±0.0 |
| Urinary β2 microglobulin (mg/L) <0.25 | 2.7±1.3 |
| Urinary κ/λ 0.7-4.5 | 0.2±0.0 |
| IgG- λ % negtaive | 100 |
| Bence-Jones λ % negtaive | 100 |
| Serum amyloid A，SAA（mg/L）＜10 | 4.8 |

AST, alanine aminotransferase; ALT, aspartate aminotransferase; ALP, alkaline phosphatase; g-GT, gamma-glutamyltransferase; hATTR-PN, hereditary transthyretin amyloidosis-polyneuropathy; normal control; sFLC, serum free light chain;


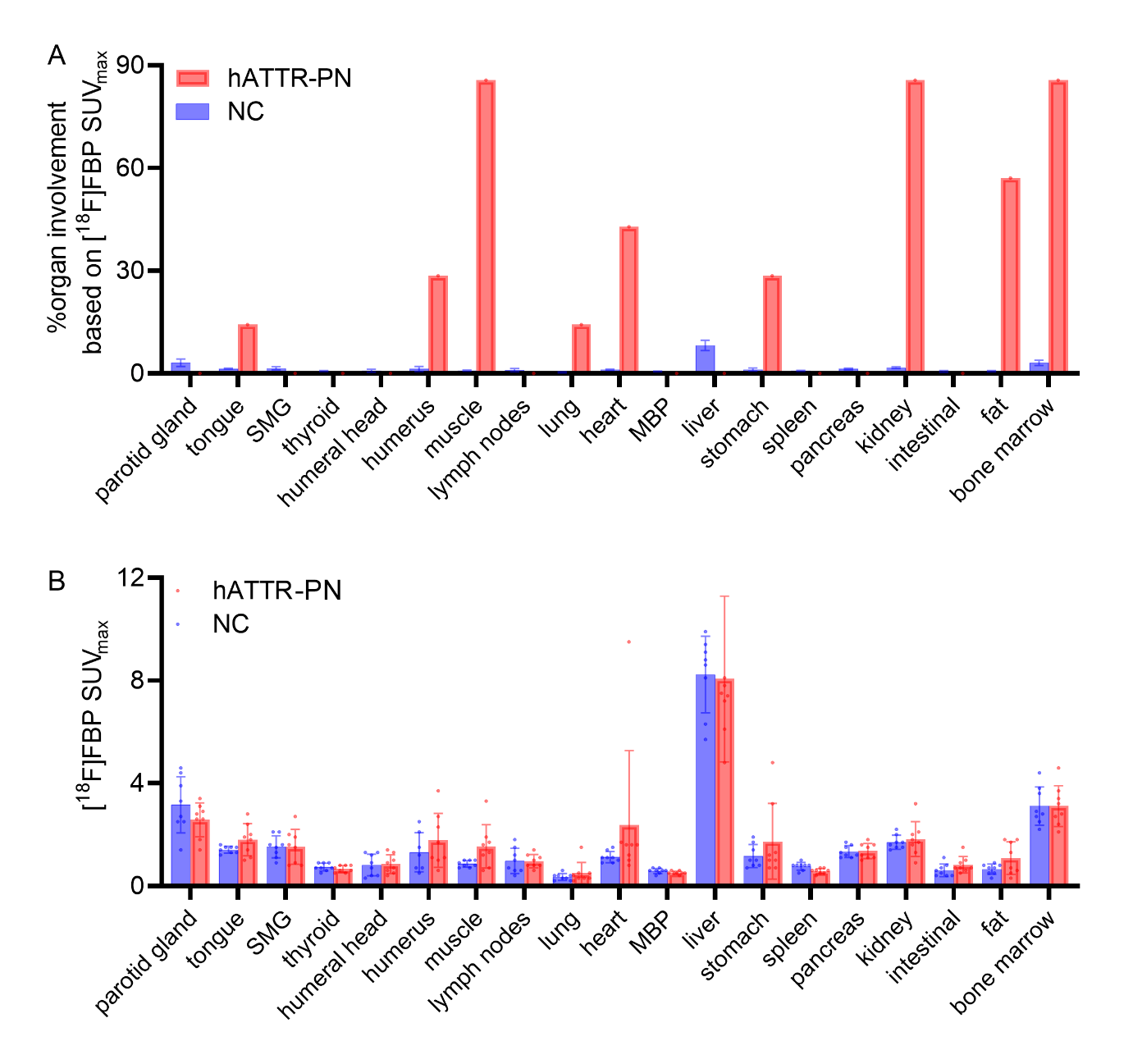


**S****Fig. 1 Regional uptake of [^18^F]FBP PET and organ involvement analysis in NC and hATTR-PN patients**. (A) Percent organ involvement according to SUVmax analysis of [^18^F]FBP PET; (B) [^18^F]FBP SUVmax analysis in NC and hATTR-PN patients; mediastinal blood pool (MBP); submandibular gland (SMG).


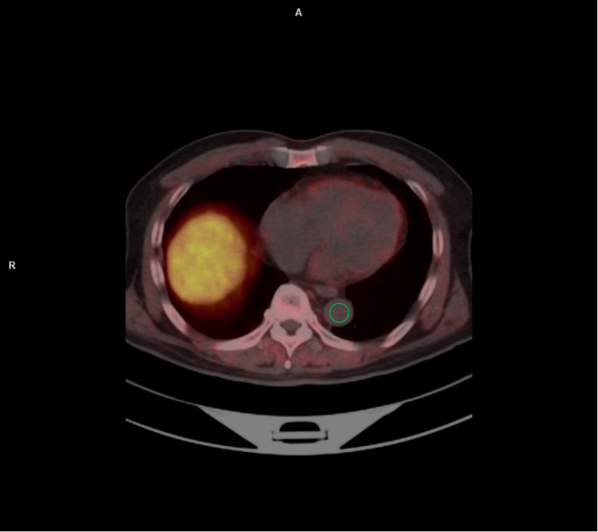


**SFig. 2 Reference region for the TBR analysis of the [^18^F]FBP PET**. The diameter of the mediastinal blood pool region of interest is 1 cm (green circle).

# **Methods**

**Patient information**

The participants volunteered to join the study cohort through an advertisement on a public board. Further cardiac ultrasound amyloid screening and bone marrow (BM) examinations were performed. If the results of the patient's serum test were abnormal and clinical manifestations of suspected systemic amyloidosis, such as heart failure, elevated proBNP and cTnT, low voltage on the electrocardiogram, proteinuria, elevated ACR and PCR, renal impairment, liver damage, nerve damage, especially axonal damage, skin manifestations, tongue hypertrophy, or muscle damage, were detected, all participants underwent [^18^F]FBP PET/computed tomography (CT).

**Radiosynthesis**

The [^18^F]FBP tracer was prepared in the Department of Nuclear Medicine, PET Center, Huashan Hospital, Fudan University, as described earlier. [^18^F]FBP (0.56 GBq/ml) was radiosynthesized from its precursor via a fully automated procedure [1, 2].

**PET/CT**

[^18^F]FBP PET/CT was conducted (Biograph Truepoint HD 64 PET/CT or Biograph mCT Flow, Siemens, Germany) with previously described parameters [1]. For [^18^F]FBP PET, the subjects were intravenously injected with 0.37-0.55 MBq/kg [^18^F]FBP. At 50 min after the injection, a 2‐min static PET acquisition for the body in 3D+time of flight (TOF) mode with a 12.18 s CT scan (120 kV, 150 Ma) for attenuation correction was performed [3]. After the scan, a 10‐min static positron emission tomography (PET) scan of the brain was performed in 3D+TOF mode with a 12.18 s CT scan (120 kV, 150 Ma) for attenuation correction. After acquisition, all the brain PET images were reconstructed via a backprojection + TOF algorithm with the following parameters: image size= 256×256, Gaussian filter, zoom factor 2, and full width at half maximum (FWHM) = 3.5 [1]. For whole-body PET, images were reconstructed via a Gaussian filter, full width at half maximum (FWHM) = 4, zoom = 1, image size = 200×200, recon method Trux+TOF, iteration = 4.

**Image analysis**

Two experienced PET specialists used the binary visual reading method to analyze the involved organs on [^18^F]FBP PET images. When the evaluations of the two PET specialists were not consistent, a senior PET specialist provided further evaluation. The positivity of the PET scans in both the brain and the periphery was visually analyzed. Standard uptake value (SUV) and target-to-background ratio (TBR) analyses of [^18^F]FBP PET data were performed for 20 organs, including the parotid gland, tongue, submandibular gland, thyroid, humeral head, humerus, muscle, lymph node, lung, heart, mediastinal blood pool (MBP), liver, stomach, spleen, pancreas, kidney, intestine, fat, and BM. Bladders were not included in the analysis because urine accumulation confounded the results. Since only one patient showed positive uptake in the spinal cord, retina, breast and skin, we did not perform an analysis of these organs. Volume-of-interest measurements of SUV_max_ were obtained. For most organs other than the liver, [¹⁸F]FBP uptake was considered positive if the SUV exceeded the cutoff value of 2.5, as established in [4]. For organs with inherently low [¹⁸F]FBP background uptake (e.g., fat), a focal hotspot above background was deemed sufficient for positivity. The TBR was computed using the MBP (circle diameter of 1 cm, **SFig. 2**) as the reference region for both [^18^F]FBP PET.

MBP was selected as the reference region for TBR calculations to minimize confounding effects from physiological uptake in the liver and heart. MBP was preferred due to its well-established physiological stability, ease of measurement, and reliability as a stable background reference in PET quantitative analysis. [^18^F]FBP is excreted via both the gastrointestinal and urinary tracts, leading to notable physiological uptake in organs such as the kidneys, bladder, and gastrointestinal system. To minimize measurement errors, circular regions of interest (ROIs) with a fixed diameter of 1 cm were used for MBP. Furthermore, ROIs were delineated across at least three adjacent slices, with averaged values used to enhance measurement consistency and reduce variability.

**Biochemical analysis and staining of biopsy tissues**

Biochemical analysis (serum, urine) was performed for indicator reflecting the status of different organs. In the liver, alanine transaminase (ALT), aspartate transaminase (AST), gamma-glutamyl transferase (r-GT), alkaline phosphatase (ALP), and total bilirubin were measured following standard procedures. For the heart, cardiac troponin T (cTnT), N-terminal pro-B-type natriuretic peptide (NT-proBNP), heart-type fatty acid-binding protein (hFABP), and serum free light chain (SFLC) were measured following standard procedures. For the kidney, the estimated glomerular filtration rate (eGFR) was measured following standard procedures. For urine, the urine protein, kappa and lambda light chains, as well as the Bence-Jones protein, were measured following standard procedures.

Genetic testing was performed for participants with ATTR to confirm mutations (xymedlab, Genery Biotechnology, Amcarelab, and Kangso Medical, China). A blind test of abdominal wall fat biopsy or rectal biopsy was performed if not available. Biopsies were collected from the retina, muscle, liver, fat, skin, liver, kidney, bladder, lung and brachial plexus. The biopsy site was determined on the basis of the imaging and clinical examination results. The site of amyloid deposition was used if available. Congo red staining and hematoxylin and eosin (H&E) staining were performed on the paraffin-embedded fixed tissue biopsies following standard procedures. Kappa and lambda immunofluorescence staining were performed on frozen tissue biopsy sections following standard procedures. The stained sections were scanned at ×20, ×40 and ×100 magnifications using a Nikon anti-mould microscope (551 DS-F1, Nikon Instruments). Images were adjusted using ImageJ (NIH).

**Statistics**

Statistical analysis was performed using GraphPad Prism 10 (GraphPad). The nonparametric Mann‒Whitney test was used for comparisons between two groups. Two-way ANOVA was performed with Sidak’s post hoc test. Correlations between different readouts were analyzed by using nonparametric Spearman’s rank analysis. The values are expressed as the means ± standard deviations. The significance level was set at p<0.05.

**References**

[1] J. Wang, Q. Huang, K. He, J. Li, T. Guo, Y. Yang, Z. Lin, S. Li, G. Vanderlinden, Y. Huang, K. Van Laere, Y. Guan, Q. Guo, R. Ni, B. Li, F. Xie, Presynaptic density determined by SV2A PET is closely associated with postsynaptic metabotropic glutamate receptor 5 availability and independent of amyloid pathology in early cognitive impairment, Alzheimers Dement (2024).

[2] Y. Kong, C.A. Maschio, X. Shi, F. Xie, C. Zuo, U. Konietzko, K. Shi, A. Rominger, J. Xiao, Q. Huang, R.M. Nitsch, Y. Guan, R. Ni, Relationship Between Reactive Astrocytes, by [(18)F]SMBT-1 Imaging, with Amyloid-Beta, Tau, Glucose Metabolism, and TSPO in Mouse Models of Alzheimer's Disease, Mol Neurobiol (2024).

[3] S. Minoshima, A.E. Drzezga, H. Barthel, N. Bohnen, M. Djekidel, D.H. Lewis, C.A. Mathis, J. McConathy, A. Nordberg, O. Sabri, J.P. Seibyl, M.K. Stokes, K. Van Laere, SNMMI Procedure Standard/EANM Practice Guideline for Amyloid PET Imaging of the Brain 1.0, J Nucl Med 57(8) (2016) 1316-22.

[4] E.C. Ehman, M.S. El-Sady, M.F. Kijewski, Y.M. Khor, S. Jacob, F.L. Ruberg, V. Sanchorawala, H. Landau, A.J. Yee, G. Bianchi, M.F. Di Carli, R.H. Falk, H. Hyun, S. Dorbala, Early Detection of Multiorgan Light-Chain Amyloidosis by Whole-Body (18)F-Florbetapir PET/CT, J Nucl Med 60(9) (2019) 1234-1239.
